# Binary-trans, non-binary and gender-questioning adolescents’ experiences in UK schools

**DOI:** 10.1080/19361653.2021.1873215

**Published:** 2021-01-19

**Authors:** Susie Bower-Brown, Sophie Zadeh, Vasanti Jadva

**Affiliations:** Centre for Family Research, University of Cambridge, Cambridge, UK

**Keywords:** Transgender, non-binary, gender-questioning, school, adolescence

## Abstract

A growing number of adolescents are using a number of different identities to describe their gender. Schools have been noted for their uninclusive environments and high levels of discrimination for LGBT + individuals, yet research has neglected the school experiences of UK gender-diverse adolescents. This article explores the school experiences and navigation strategies of gender-diverse adolescents in the UK, examining the experiences of binary-trans, non-binary and gender-questioning adolescents separately. The data presented in this article come from a large survey of LGBTQ + young people’s social experiences; a subsample of 74 adolescents’ (25 binary-trans, 25 non-binary, and 24 genderquestioning) open-ended responses were selected for qualitative thematic analysis. Findings highlight that gender-diverse adolescents experience discrimination within the school environment from the curriculum, space, peers and teachers, and a number of strategies, including disclosure negotiation, cognitive restructuring and proactive protection, are used to navigate this environment. Findings shed light on the school experiences of gender-diverse adolescents, and suggest that the British school system is fundamentally unsuitable for nonbinary and gender-questioning identities.

## Introduction

More and more young people in the UK do not identify with the gender that corresponds to the sex-category they were assigned at birth (GIDS 2018).^1^ Schools have been highlighted as the place where gender-diverse^2^ adolescents face the most discrimination (LGBT Youth Scotland, 2018; Wyss, 2004); given the prominence of the gender binary throughout the school day (e.g. uniforms, toilets, changing rooms, sport), and the large proportion of time that adolescents spend at school, understanding the experiences of gender-diverse adolescents in this context is important. Moreover, given that an increasing number of gender-diverse adolescents are identifying as non-binary (LGBT Youth Scotland, 2018), understanding how school experiences differ among those with different gender identities is significant. This article therefore asks three questions: what are the school experiences of binary-trans, non-binary and gender-questioning ado-lescents in the UK? How do binary-trans, non-binary and gender-questioning adolescents navigate the school context? And do experiences and strategies differ between these three groups? Broadly, it seeks to understand the experiences of gender-diverse adolescents in UK schools.

Research has consistently shown that gender-diverse youth experience worse mental health than their cisgender counterparts (e.g. Eisenberg et al., 2017), with research finding an association between perceptions of school connectedness and mental health outcomes, including self-harm and suicide attempts (Veale et al., 2017). Studies have reported high rates of bullying for gender-diverse youth, finding an impact of bullying on adolescents’ self-esteem and academic achievement (Day et al., 2018; Wyss, 2004). However, it is important not to focus only on bullying at the expense of discussions about underlying cisheternormativities within schools, and to recognize institutional forms of cisgenderism, that is, the pervasive ideology that pathologises and delegitimises non-cisgender identities (Ansara & Hegarty, 2012; Gilbert et al., 2018). Researchers have also highlighted that gender-diverse youth are affected by oppressions beyond cisgenderism, including racism, classism and ableism (Gill-Peterson, 2018). Moreover, multiple aspects within the school environment have been found to affect the outcomes of gender-diverse adolescents – for example, lack of teacher support increases the likelihood of gender-diverse students leaving school (Jones et al., 2016) and lack of choice in bathroom and changing room use has been found to be associated with an increased likelihood of gender-diverse youth experiencing sexual assault (Murchison et al., 2019). Such findings point to the importance of studying multiple aspects of adolescents’ school experiences, including peers, teachers, and provision of spaces, and studying how adolescents navigate this cisheteronormative environment (Travers, 2018).

Research on gender-diverse adolescents’ school experiences within the UK is overall lacking. The UK environment is hostile to gender-diverse youth: schools with LGBTQ+ initiatives, such as inclusive curricula and rainbow zebra crossings, have been targeted by protests (Busby, 2020; Parveen, 2019); sociolegal debates about access to puberty blockers for gender-diverse youth are ongoing (Holt, 2020); and provision of gender-neutral spaces are currently the subject of political debate (O’Reilly, 2020). However, little is known about the experiences of gender-diverse youth within this social and political context. One recent UK-based study focused on the experiences of parents who support their gender-diverse children, and found that parents are supported by schools on an ad-hoc basis, in the absence of proactive policies (Davy & Cordoba, 2020). Given the absence of such policies, and the hostile social and political climate, finding out about the school experiences of gender-diverse youth in the UK – as they themselves describe them – is both timely and important.

This article reports findings from a study investigating the school experiences of binary-trans, non-binary and gender-questioning individuals within the UK. In so doing, it seeks to move beyond previous research that has tended to focus on, and privilege the narrative of, binary-trans individuals (Frohard-Dourlent et al., 2017). Most of the literature to date on the school experiences of gender-diverse adolescents has tended to either include non-binary and gender-questioning individuals under the trans umbrella, or omit them completely, and it is often unclear whether or not they have been included. Some research has explored potential differences in the experiences of binary-trans and non-binary youth: studies have found that binary-trans youth report more hostile school experiences and lower life satisfaction than non-binary youth (Kosciw et al., 2018; Rimes et al., 2019); whilst others have found that non-binary youth receive less social support and experience higher levels of anxiety and depression than binary-trans youth(Aparicio-García et al., 2018; Thorne et al., 2019). Non-binary students may find it difficult to socially transition due to a lack of societal understanding around non-binary identities, in the context of media positioning of such identities as ‘other’ (Frohard-Dourlent, 2018; Richards et al., 2016). Research with non-binary US college students has highlighted the burden on non-binary students to be a ‘gender educator’ in the face of this misunderstanding (Goldberg & Kuvalanka, 2018). Alongside the mixed findings on the experiences of non-binary adolescents, there is no research examining the potentially unique experiences of gender-questioning adolescents at school. Overall, research on gender-diverse youth’s school experiences has tended to be based on samples with parental support, as parents often act as gatekeepers to research participation (e.g. Davy & Cordoba, 2020; Travers, 2018). This study reports on the experiences of a sample of gender-diverse youth who did not require parental consent to take part, and therefore may have varying levels of family support.

## Methods

### Sample

The data for this article come from a large survey of LGBT young people, conducted in collaboration with Stonewall. The survey was available online and on paper and was open to young people (aged 11-19) who identified as LGBT and lived in England, Scotland or Wales. Importantly, a waiver of parental consent was granted to protect young people who had not disclosed their sexual orientation and/or gender identity to parents or guardians, allowing the inclusion of adolescents without family recognition or support. This study was granted ethical approval by the University of Cambridge Psychology Research Ethics Committee. Data were collected from November 2016 – February 2017. The survey consisted of closed and open-ended questions. This article focusses on the open-ended questions that asked about school experiences.

A subsample of this survey is focused on in this article: survey responses were excluded if respondents identified as cis, were aged 11-12 or 18-19, or answered less than half the open-ended questions. The responses of youngest and oldest participants were removed so as to focus on school rather than university, and to focus only on adolescence; those who had answered less than half of the open-ended questions were removed, so that there would be sufficient data for qualitative analysis. Participants were then grouped into three broad categories of gender identity (binary-trans, non-binary and gender-questioning) based on their responses to the questions “*are you trans?*” (yes/no option) and “*what is your gender?*” (male, female, or open-ended option). Participants were categorized as binary-trans if they answered ‘yes’ to being trans and gave their gender as male or female, or described their gender as binary-trans (e.g. ‘trans boy’, ‘trans girl’); participants were categorized as non-binary if they gave their gender as something other than male or female (e.g ‘agender’, ‘genderfluid’, ‘genderqueer’); participants were categorized as gender-questioning if they answered ‘unsure/questioning’ to being trans, ‘unsure/questioning’ to the question about their gender, or gave a response that indicated uncertainty (e.g. ‘unsure/questioning girl’, ‘non-binary but unsure’, ‘possible MtF trans’). Once the categories had been constructed, participants were selected using stratified random sampling to ensure an even spread of ages and gender identities. 5 binary-trans participants each aged 13, 14, 15, 16, and 17 were selected and the same approach was taken to selecting the non-binary and gender-questioning participants. This resulted in a final sample of 25 binary-trans, 25 non-binary and 24 gender-questioning participants, as only 4 (not 5) gender-questioning participants aged 16 met the criteria for inclusion.

It is recognized that categorizing people who may see their gender as uncategorisable is potentially problematic. The three categories have been created not to make any claims about inherent differences between the categories, but rather to explore any potential differences in school experiences. It is important to recognize that ‘binary-trans’, ‘non-binary’ and ‘gender-questioning’ are not static or discrete categories; their meanings change across time and context. However, they can be seen as conceptual tools with which to explore adolescents’ experiences of the gender binary (Goldberg & Kuvalanka, 2018). Equally, creating additional categories under the ‘gender-diverse’ umbrella overcomes issues with previous research, where non-binary and gender-questioning individuals have been neglected. Participants’ identities and individualities are described in rich detail throughout the results section and categorization, therefore, has not been used to reduce participants to their gender identities, but to facilitate breadth and richness of analysis, and reflect the heterogeneity of genderdiverse adolescents.

### Data analysis

The data were analyzed according to the principles of thematic analysis and thematic coding (Braun & Clarke, 2006; Flick, 2014), which, when used together, broadly correspond to a qualitative comparative approach (Guest et al., 2014). The aims of qualitative comparisons are to establish whether themes and subthemes exist in all groups under study, and where themes and subthemes are present in multiple groups, to establish the similarities and/or differences in their expression between groups. Following this approach, the data of the three participant groups were analyzed separately using Atlas.ti, and initial line-by-line coding resulted in the generation of 240 codes across the binary-trans data-set, 260 codes across the non-binary data-set, and 211 codes across the gender-questioning data-set. Some codes existed across the three groups (e.g ‘staff ignore anti-LGBT language’), whereas others were specific to each group (e.g. ‘doesn’t fit into the binary’ was only relevant to non-binary participants).

Each of the code lists were then shortened, refined and organized into themes and subthemes (Guest et al., 2014). The analytical process was both deductive and inductive: although data driven, the subthemes and themes also reflect the theoretical and reflexive positioning of the authors. For example, in the process of theme creation, the first author reviewed all codes through the lens of structural symbolic interactionism (Stryker, 1980), as this theory highlights the way in which identity exists within interaction. Regular meetings were also held during the process of theme development, partly serving as a space to reflect upon the role of the authors’ own experiences and identities in the analytical process (Mccorkel & Myers, 2003). The authors are cisgender researchers, and although it is recognized that it would not have been possible to account for all cisnormative assumptions, the authors aimed to engage with their own positionality and the *process* of analysis throughout (Braun & Clarke, 2019; Vincent, 2018). This approach included, but was not limited to, taking trans awareness training; partnering with an LGBTQ organization; engaging mindfully with language; and describing participants’ identities and experiences in their own words throughout the results section.

## Results

The results section answers three research questions: 1. What are the school experiences of binary-trans, non-binary and gender-questioning adolescents in the UK? 2. How do binary-trans, non-binary and gender-questioning adolescents navigate the school context? 3. Do these experiences and strategies differ between the three groups? Three themes were found to relate to the first question: normativities; space; and peers and teachers. Similarly, three themes were found to relate to the second question: disclosure negotiation; cognitive restructuring; and proactive protection. In relation to the third question, the themes and subthemes were found to be relevant to the three groups of participants in varying degrees of magnitude and meaning. Where present, such differences are expanded upon below.

### School experiences

Participants noted that they experienced high levels of discrimination within the school environment, described below in the subthemes of normativities, space, and peers and teachers.

### Normativities

Across the dataset, participants explained that a number of normativities characterized their experiences with school. For example, participants described the role that cisheteronormativity played in language, the school curriculum, and policy:

“My school is so lenient with the usage of slurs like ‘faggot’ and ‘tranny’ that students feel comfortable enough to use them in their regular vocabulary, even in lessons to answer questions” (magiboy, aged 13).

“I have never been taught in school about LGBT issues until anti-bullying week, where the focus was apparently LGBT but nothing was done except one assembly that instead focused on ‘tolerance’ as a whole and didn’t even mention LGBT issues” (unsure person, aged 16).

Across the dataset, participants suggested that if LGBTQ + issues were mentioned in their school, education was mostly limited to “mild homophobia” (trans boy, aged 14): “there was an epidemic of homophobic slurs that got ironed out pretty fast but they never did anything against transphobia” (trans girl, aged 16).

Non-binary participants reported pressure to exist within the gender binary: “my school is very binary and I feel I can’t come out at school as they wouldn’t know what to do with me” (agender person, aged 14). Another participant highlighted school policy as being predicated on a stable binary: “they refused to let me present the way I identify and refuse to let me use whatever toilet I would like” (agender person, aged 16). This highlights the unique experiences of non-binary participants. Binary-trans participants did not mention the restrictive nature of the gender binary, however, they expressed facing difficulties, for instance in relation to their schools’ interpretations of UK law and its implications for school practice:

“[The school] would only let me use my preferred name if I got it legally changed to that” (trans boy, aged 17).

“I really want to have PE with the other boys but I’m stuck with the girls because it’s the law” (trans boy, aged 14).

These experiences suggest transnormativity within the school environment, meaning the way in which gender-diverse individuals are held accountable to a binary, medical model of transness (Johnson 2016). Here, participants’ experiences suggest that schools may allow inclusion of binary-trans individuals, but only when backed up by ‘legal’ determinants of gender, although there are no such requirements on schools in law. Importantly, non-binary identities are not legally recognized within the UK, meaning that such ‘legal’ means could not be used by many of the participants in this study to ensure respect of their gender identity at school.

### Space

Alongside the role of normativities in affecting participants’ experiences, participants across the dataset described experiencing difficulties in terms of physical space in school, although participants’ experience of space differed. Binary-trans participants were often able to access alternative spaces (“I also use the accessible toilet to get changed in at school for P.E. to not assign me to a gender or upset me or others around.” (trans male, aged 14)), but some expressed feeling a sense of otherness due to the unsuitability of these spaces:

“I can use the female staff changing room which makes me kind of uncomfortable because people stare at me when I go in or out” (trans boy, aged 14).

Non-binary young people expressed a desire for gender-neutral spaces, as these were not provided (“it would really help if we had unisex bathrooms” (non-binary person, aged 15)), and described feeling policed by other students for using gendered facilities (“told I’m using the wrong bathroom” (non-binary person, aged 15)). One participant also described how school policy contradicted their wishes: “they wanted me to find a disabled toilet (only one available and it’s all the way in the sixth form that wasn’t always available) to get changed in despite me saying I was fine in the female changing room” (non-binary person, aged 15). These experiences suggest a policing of non-binary students’ gender by staff and students, potentially highlighting a lack of space for non-binary identities. Genderquestioning participants also described the challenges posed by gendered spaces, and this was partly due to their own uncertainty about which facilities to use:

“I used to have a running streak of weeks I would cry after PE because I was stuck in the girls changing rooms and be with the girls, but it is probably better with the girls than with the boys” (gender fluid/questioning person, aged 14).

One could interpret this as a lack of metaphorical and physical space for uncertainty in the social environment, which gender-questioning participants felt to be particularly restrictive:

“I find it impossible to experiment with my gender identity (dressing and acting like a girl, using a female name etc.) without coming out” (possible MtF trans, aged 17).

The themes of normativities and space therefore highlight the ways in which the school environment was restrictive for all participants in this study. For binary-trans participants, this led to a sense of otherness; non-binary participants felt unable to exist within binary spaces; gender-questioning participants not only felt uncertain about which space they should occupy but also felt restricted by the lack of space for uncertainty in their gender identity and expression.

### Peers and teachers

Participants expressed that peers’ and teachers’ reactions to their identity were important in determining their school experience. Across the dataset, participants reported a lack of societal understanding about gender diversity, and described receiving negative reactions as a result. Binary-trans participants found that this lack of understanding (“no one understands what being transgender is so it make me feel terrible when people make jokes” (trans boy, aged 13)) took the form of bullying:

“A girl in my primary school bullied me verbally and psychologically for 6 years. She tried to tell me how to be a better girl even though I am a trans boy.” (trans boy, aged 13).

Due to this bullying, binary-trans participants described emotions ranging from annoyance (“sometimes it annoys me” (trans boy, aged 13)) to anxiety (“because of this bullying I now have a phobia of having videos or pictures taken” (trans boy, aged 13). Bullying also affected participants’ behavior, here in terms of moving school for the last two years of compulsory education: “It’s the reason I chose to go to a college instead of staying on for sixth form at my current school” (trans girl, aged 16).

Non-binary and gender-questioning participants also reported negative reactions to their identity:

“Someone from school found [my anonymous twitter account] and spread rumours, after that people always commented on my small boobs, short hair, lack of makeup, until it got so bad I hated my body, the uniform I had to wear” (both gender person, aged 16).

“I am now being bullied not as severely by a group of girls who make comments about my body and try to out me in several lessons but I have too much anxiety around telling anyone about my experience” (unsure/questioning girl, aged 15).

This bullying seemed to be particularly impactful for non-binary participants (“Scared. That’s the best way I can describe it. I’m scared of being abused for who I am” (demigender/demigirl, aged 14)) and gender-questioning participants (“it makes you feel like you want to dig a hole into the ground and stay there for the rest of your life” (unsure/questioning girl, aged 14)). This potentially demonstrates the challenge of having an identity that is not legally recognized, or questioning an identity in an environment that relies on gender stability. As one participant said, “… in their eyes, you’re either cis, trans or intersex” (gender questioning person, aged 15). This also affected participants’ future expectations: “past bullying has made me scared about my future because I’m scared that no one will want to hire me because of my gender identity” (agender person, aged 16).

These participants’ experiences suggest that whilst there may be limited understanding of binary-trans identities within the school context, the same cannot be said of non-binary and questioning identities:

“I did one time tell a close friend about me questioning my gender … who told a friend … and she confronted me about it and assumed I ‘wanted to have a dick’” (gender questioning person, aged 17).

It is worth noting that research on LGBTQ + youth’s social experiences has been focused primarily on interactions with peers; in the present study, participants expressed that teachers had a unique authoritative power to either help or hinder them. A minority of participants suggested that teachers were “helpful with dealing with other students who misgender me” (trans boy, aged 17). However, most participants described finding teachers unhelpful or even engaging in bullying behaviors themselves:

“Some staff are okay with me being trans but a minority say insults and use the wrong pronoun on purpose. When I complain about being insulted by staff to other teachers they say that everyone’s entitled to their own opinions.” (trans boy, aged 14).

“Sometimes teachers actually laugh at the comments or don’t do anything which is really upsetting and makes me anxious as someone who doesn’t identify as their assigned gender.” (non-binary but unsure person, aged 16).

Teachers’ lack of understanding and bullying behaviors were therefore described as reifying and legitimizing anti-LGBTQ + bullying within school:

“Attending a school with an openly homophobic and transphobic senior staff team, that greatly affected my schoolwork and feeling safe at school as I felt if anything did happen to me, nothing would happen” (unsure/questioning girl, aged 17).

### Navigation strategies

As seen above, participants described feeling excluded by the school environment. They also described using a range of strategies to navigate this environment: disclosure negotiation, cognitive restructuring, and proactive protection.

### Disclosure negotiation

All participants constantly negotiated disclosure of their identity, and the complexity of this negotiation was a key strategy for navigating the school environment for binary-trans, non-binary and gender-questioning participants. Negotiating disclosure was found to be “a continual process” (trans girl, aged 17), with participants coming out multiple times, in a number of ways, including online (“I came out as FtM trans on Instagram” (trans boy, aged 14)), or through humor (“some form of joke or sarcastic comment” (unsure/questioning girl, aged 17)). However, participants also reported that it was not always possible to control information about their identity: “Someone had outed me after I fell out with him and lots of people started being rude about my gender and it was intimidating and pretty scary” (non-binary but unsure person, aged 16). Such findings are particularly important when considering participants’ concerns about safety:

“If I out myself at school I will be forced to change schools because I know I would be relentlessly bullied … I’m afraid that if I make one wrong move I will end up outing myself to everyone” (magiboy, aged 13).

Participants also felt that the fear of being unsafe distanced them from the potential benefits of coming out (“it would make my life so much easier, but I have been put off by the lack of support and education about LGBT + issues” (trans boy, aged 14)), including mental health support (“I feel like I can’t tell anyone that I have self-harmed. If I do, they will want to know the reasons why but I can’t risk coming out to anyone” (magiboy, aged 13)). Therefore, lack of school support for gender diversity meant that participants did not feel able to come out, and this was experienced as very stressful.

For gender-questioning participants, disclosure negotiation was further complicated by their own identity-related uncertainty:

“It’s frustrating that no one calls me he or by my preferred name, but at the same time I don’t want them to because I don’t want others to know, especially as I’m still questioning.” (unsure/questioning girl, aged 16).

“I find it possible to experiment with my gender identity … I want to transition fully into a woman (once I am 100% certain of my gender).” (possible MtF trans, aged 17).

These findings highlight the ways in which the lack of space for gender fluidity appeared to impact upon the strategies that gender-questioning adolescents use. Gender-questioning participants also highlighted that their uncertainty restricted them from others’ support:

“Some people doubt that I’ll go through with fully coming out and others don’t use my preferred name in the school environment, for fear of being questioned” (unsure/ questioning girl, aged 13).

Relatedly, some participants described feeling uncertain about whether existing social categories fully described their experience:

“When I said that I might be trans, I meant that I’m a bit non-binary, but I don’t know whether it’s to the extent that I would take action on it.” (unsure/questioning boy, aged 17).

“It’s difficult coming out as trans and as bi as I feel like it’s seen as un-manly and that I’m not properly trans” (trans boy, aged 17).

Participants’ uncertainty about their identity or being ‘trans enough’ highlights transnormativity in participants’ experiences. Disclosure negotiation was therefore an important tool for navigating the school environment, but was also made more complicated for gender-questioning participants, highlighting the importance of focusing on their unique experiences.

### Cognitive restructuring

Across the dataset, a number of participants used cognitive strategies in their responses to negative social experiences. This has been termed ‘cognitive restructuring’ as it describes how participants conceptually framed their experiences in order to minimize the impact of negative experiences on the self. For instance, some adolescents perceived a difference between their authentic self and the self they presented to the world, ultimately serving to protect their true self:

“Socially I only make friends with & date other LGBT people who live in other cities … I love getting to be me when I go to Brighton and not caring what anyone else thinks.” (queer person, aged 14).

“In the real world I remain very much in the closet … I know that one day I will be ready, and I hope I will, but for now I will remain where I am” (non-binary person, aged 14).

These quotations highlight the potentially protective nature of an authentic self, different from that which is found at school. Some participants denied negative school experiences and their effects. Although it is vital to allow space for positive experiences, the ambivalent narratives found among these participants seem to indicate that such disavowal may function as a coping strategy. For example, one non-binary participant wrote:

“After coming out to the people around me, I was taunted a lot … It didn’t really affect anything as I didn’t take it to heart and carried on with my life as normal apart from switching back to my given name” (gender non-binary person, aged 13).

Elsewhere in the survey this participant also wrote: “I think your preferred name and pronouns should be used”, potentially suggesting an earlier understatement of the effects of identity threat. Another participant denied that being trans had affected their life: “I don’t feel the need to surround myself with other LGBT to be accepted and comfortable. School is good. Home is good. Being trans hasn’t affected my life much at all.” (trans boy, aged 15). However, the same participant also noted “Hari Nef is good trans role model … Frank Ocean wearing make-up is also quite good”, thus reflecting an interest in LGBTQ + role models. They also described being “too nervous to use the boys’ toilets”, potentially suggesting that anxieties about difficult social experiences may not always be explicitly articulated as such. Other participants used classism to discredit the people who threatened them: “the chavs at school” (queer person, aged 14) or “the rougher kids who tend to be the ones who use [homo/bi/transphobic] language” (trans boy, aged 14). Such a strategy has been previously identified in the literature as condemning the condemners (Sykes & Matza, 1957), a means by which stigmatized individuals may limit the effect of discrimination on the self.

### Proactive protection

Across the dataset, proactive protection was a strategy commonly used by participants, and this strategy was described as being particularly effective. Proactive protection included seeking out LGBTQ + people and allies, creating communities, and engaging in activism. One participant noted about their school experience, “it sucks, but I try to remain proud” (genderfluid person, aged 17). Participants across the dataset also noted that their friendships with other LGBTQ + students were particularly important for their identity:

“It’s funny, we all grouped together before we knew we were LGBT, and then we all gradually realised and came out one at a time … My friends coming out was a really reaffirming experience. Seeing that other people treated them ok was nice” (egogender person, aged 16).

Friends were often seen as the most positive relationships for participants, described by one participant as a “second family who make me feel normal and loved” (unsure/questioning girl, aged 17), enabling participants to overcome negative experiences:

“Sometimes [bullying] annoys me but I know that I have friends who support me and that I can go further in life than my bullies” (trans boy, aged 13).

Equally, having other LGBTQ + friends meant that some participants felt they had to challenge bullying: “As someone with a gay friend, a lesbian friend, 2 bi friends, a trans friend and some very strong opinions I stick up for all of them” (demigender/demigirl, aged 14). Participants in all three groups also described being motivated to improve the school environment for themselves and others:

“I am currently talking to my school about becoming more diverse with students and talking about mental health and also LGBTQ + issues in school.” (trans girl, aged 16).

“I wore a skirt and my science teacher asked me to stay behind after class and said how boys might think its ok for them to come to school in skirts if I was wearing one. This is now different and I’m allowed to wear both skirt and trousers” (non-binary person, aged 15).

Some participants were therefore able to undertake activism within the school, in order to protect both themselves and other LGBTQ + students. However, for some participants the burden of being a ‘gender educator’ (Goldberg & Kuvalanka, 2018) was especially heavy:

“The group of people sitting next to us were pretty popular, and I heard one of them start calling another one a “tranny”. I turned round to them and politely explained that what they said was transphobic and they shouldn’t say it, and they all laughed at me and gave me dirty looks for weeks.” (trans boy, aged 14).

“I spoke to a teacher last week who tried to justify using gay as an insult. Though they were willing to listen to me explain how using it as a negative implies you believe there is something wrong with being gay” (nonbinary/genderfluid/agender person, aged 17).

Such attempts to educate others were deemed necessary in an unsupportive environment, adding to the stress experienced by participants at school. A minority of students did experience a supportive school environment, and their responses showcased the potential for educators to construct such an environment:

“The school is amazing with homophobia, biphobia, transphobia, panphobia and many, many more … We have a club where we sometimes don’t even talk about LGBT and the amazing LGBT teacher that runs it brings us cookies and we just sit in a room and talk about anything … I absolutely love it!” (unsure/questioning girl, aged 13).

In the absence of such an environment, participants engaged in activism – one participant, for example, noted that pupils could go to “other students for advice, as they are too afraid to talk to staff” (unsure/questioning girl, aged 13). Indeed, another participant highlighted the lack of inclusion for gender diversity in the absence of students coming out:

“No one there has ever come out as trans or non-binary so no idea how the school would have handled it … they definitely would have made them wear the uniform relating to their biological sex” (unsure/questioning girl, aged 17).

Therefore, whether inclusive or exclusionary, school policy was described as directly impacting the experiences of participants. It is worth noting that gender-questioning participants noted less proactive protection than non-binary or binary-trans participants, including a lack of social support (“In my online school I have no friends I feel completely isolated” (unsure/questioning girl, aged 15)) and a lack of role models (“I’m not aware of any such role models” (unsure/questioning boy, aged 17)). This potentially suggests that due to uncertainty about their own identity, gender-questioning participants may struggle to access LGBTQ + resources and, in the absence of students coming out, schools do not create inclusive policies:

“I am still questioning [my gender] and it is really difficult in school especially as I go to an all-girls school, I still don’t know what gender identity I am but I know that I will know, and one day I will be in a safe environment where I can come out and be accepted.” (unsure/questioning girl, aged 17).

## Discussion

This article builds upon recent research on the experiences of gender-diverse youth at school (e.g. Davy & Cordoba, 2020; Frohard-Dourlent, 2018; Jones et al., 2016; Kosciw et al., 2018; Murchison et al., 2019; Travers, 2018), and is the first to investigate the school experiences of binary-trans, non-binary and gender-questioning adolescents separately in the UK context. In line with previous research, findings demonstrate that gender-diverse adolescents experience considerable discrimination within the school environment (LGBT Youth Scotland, 2018). This study extends previous research by focusing on multiple aspects of the school environment, including space, peers, and teachers. Additionally, this study adds to the existing knowledge base by highlighting the strategies that gender-diverse adolescents use to navigate the school environment.

The implications of this study are both analytical and educational. Firstly, although much research on gender-diverse youth’s experiences has focused on similarities rather than differences within this category, this study highlights the analytical benefit of deconstructing the heterogeneous ‘gender-diverse’ category. Non-binary individuals seemed to experience higher levels of discrimination than binary-trans individuals, a finding that is perhaps unsurprising considering the absence of inclusive facilities within school environments. This is not to say that the binarytrans individuals who participated in the study did not experience discrimination; indeed it is clear that binary-trans students did experience discrimination from students, teachers and policy. However, findings potentially highlights the unique difficulties faced by non-binary students in experiencing an identity that is both legally and socially unrecognized (Goldberg & Kuvalanka, 2018), and therefore attest to the importance of exclusively focusing on their experiences. Similarly, findings suggest that gender-questioning adolescents may face difficulties due the lack of space for gender fluidity within the school environment, and that their own uncertainty in terms of how to present and define themselves may be difficult, both for themselves and others. It is noteworthy that this study is the first to explore the experiences of gender-questioning adolescents; such findings suggest a need for further research on their unique experiences.

Based on participants’ experiences, proactive protection appeared to be the most effective strategy for navigating the school environment, echoing the findings of other research that has evidenced the role that genderdiverse individuals play in improving educational institutions (Goldberg & Kuvalanka, 2018). This study’s findings also demonstrate that participants who had either social and/or institutional support found it easier to protect their identity, pointing to the importance of initiatives such as LGBTQ + groups and inclusive curricula. However, in terms of its educational implications, the findings of this study suggest that schools in the UK seem to be lacking the institutional mechanisms necessary to support gender-diversity. It is noteworthy that all participants experienced discrimination at school, and for many participants, this was not only from other students, but also from teachers, the curriculum, and unsuitable spaces. In general, findings thus highlight that cisgenderism is pervasive, echoing the importance of looking ‘beyond bullying’ in both research on this topic (Gilbert et al., 2018) and in the implementation of gender-inclusive policies and practices in schools.

It is worth acknowledging that aspects of participants’ identities other than gender could have been used to categorize the overall sample, and one limitation of this study is the lack of intersectional approach to analyses. Given that gender-diverse adolescents may be affected by multiple oppressions, future research should take an intersectional approach in order to understand how to best support gender-diverse adolescents who are also impacted by other oppressions at school, including classism, ableism and racism (Gill-Peterson, 2018; Vincent, 2018) A further limitation of this study is that its participants were selected from a larger sample on the basis of having answered more than half of the open-ended questions. Although participants were asked for both positive and negative experiences, it is possible that participants who responded to fewer open-ended questions had more neutral experiences that they deemed unworthy of comment. That said, it is important to note that this study differs from other studies on the experiences of gender-diverse youth (e.g. Davy & Cordoba, 2020; Travers, 2018) in that the participating adolescents did not need parental consent to take part. The study therefore potentially reached participants with lower levels of family support. Considering that gender-diverse youth who lack family support are more vulnerable to mental health problems (Weinhardt et al., 2019), this study thus contributes to a broader understanding of the experiences of gender-diverse youth overall.

In general, the findings of this study seem to suggest that the educational system within the UK is ill-equipped to support non-binary and gender-questioning students in particular. Fundamental restructuring of the school environment is needed to support gender-diverse youth, as current support is non-existent, individualistic or tokenistic: this echoes the findings of Davy and Cordoba (2020) with regards to schools using ad-hoc policies, and those of Meyer et al. (2016) on trans and gender-creative students becoming ‘sacrificial lambs’ at school, in the absence of proactive policies. Supportive and inclusive policies that precede adolescents’ coming out have the potential to reduce the burden on individual students. Additionally, as Jones et al. (2016) also found, staff are in a unique and influential position to ameliorate the experiences of gender-diverse youth – as such, staff education is crucial. By constructing an environment in which discriminatory behavior is discouraged, and gender fluidity and flexibility are supported, schools and educators can support gender-diverse adolescents and reduce both the level and effect of discrimination on adolescents.

## Notes

1 Although some non-binary individuals do identify as trans, a number of non-binary participants referred to in this article did not, hence the use of the umbrella term ‘gender-diverse’ rather than trans or trans*. In this article, ‘binary-trans’ refers to trans individuals identifying as male or female, ‘non-binary’ refers to individuals identifying outside the binary; and ‘gender-questioning’ refers to individuals who are questioning their gender identity. It is recognised that these categories are fluid rather than discrete.

2 The umbrella term ‘gender-diverse’, used throughout the article, is specific to its context, as it is used by relevant charitable organisations in the UK (such as Mermaids (https://mermaidsuk.org.uk/) and Gendered Intelligence (http://genderedintelligence.co.uk/)) that support young people whose gender does not correspond with the sex-category they were assigned at birth.
